# Comparative Proteomic Analysis of Visceral Adipose Tissue in Morbidly Obese and Normal Weight Chinese Women

**DOI:** 10.1155/2019/2302753

**Published:** 2019-12-18

**Authors:** Chen Shang, Wei Sun, Chunlin Wang, Xiangqing Wang, Huijuan Zhu, Linjie Wang, Hongbo Yang, Xue Wang, Fengying Gong, Hui Pan

**Affiliations:** ^1^Key Laboratory of Endocrinology of National Health Commission, Department of Endocrinology, Peking Union Medical College Hospital, Chinese Academy of Medical Science, Peking Union Medical College, Beijing 100730, China; ^2^Core Facility of Instrument, Institute of Basic Medicine, Chinese Academy of Medical Sciences, School of Basic Medicine, Peking Union Medical College, Beijing, China

## Abstract

**Objective:**

Visceral adipose tissue (VAT) plays a central role in the balance of energy metabolism. The objective of this study was to investigate the differentially expressed proteins in VAT between morbidly obese (BMI >35 kg/m^2^) and normal weight Chinese women.

**Method:**

Nine morbidly obese women and 8 normal weight women as controls were enrolled. Abdominal VAT was excised and analyzed by label-free one-dimensional liquid chromatography tandem mass spectrometry (1D-LC-MS/MS). Differentially expressed VAT proteins were further analyzed with Gene Ontology (GO) analysis and Ingenuity Pathway Analysis (IPA). Masson's trichrome staining and CD68 immunohistochemical staining of VAT were conducted in all subjects.

**Result:**

A total of 124 differentially expressed proteins were found with a ≥2-fold difference. Forty-one proteins were upregulated, and 83 proteins were downregulated in obese individuals. These altered VAT proteins were involved in the attenuation of the liver X receptor/retinoid X receptor (LXR/RXR) signaling pathway and the activation of the acute-phase response process. Three proteins (ACSL1, HADH, and UCHL1) were validated by western blotting using the same set of VAT samples from 6 morbidly obese and 7 normal weight patients, and the results indicated that the magnitude and direction of the protein changes were in accordance with the proteomic analysis. Masson's trichrome staining and CD68 immunohistochemical staining demonstrated that there was much more collagen fiber deposition and CD68-positive macrophages in the VAT of morbidly obese patients, suggesting extensive fiber deposition and macrophage infiltration.

**Conclusion:**

A number of differentially expressed proteins were identified in VAT between morbidly obese and normal weight Chinese females. These differential proteins could be potential candidates in addressing the role of VAT in the development of obesity.

## 1. Introduction

Obesity, especially morbid obesity, is now a major health problem [1]. Obesity could significantly increase the risk of several comorbid disorders, such as type 2 diabetes, cardiovascular diseases, dyslipidemia, and high blood pressure [2]. The World Health Organization (WHO) has predicted a significant decline in mean life expectancy due to obesity [3]. In Asia, for any given BMI, Asians tend to have a greater percentage of body fat and a higher cardiovascular risk [4]. Moreover, sedentary lifestyles and changes in the traditional diet are among the many risks proposed for the newly acquired weight problem of Asians. China, once considered to have one of the leanest populations in the world, has experienced rapidly escalating rates of overweight and obesity [4]. Thus, it is important to elucidate the obesity-related pathophysiological alterations in Chinese individuals and to explore novel antiobesity targets.

For some obese patients, weight-loss surgery has provided therapeutic success, resulting in significant weight loss outcomes and a reduction in weight-associated comorbidities compared to the effects of nonsurgical interventions. Females are the predominant gender to receive bariatric procedures, especially adjustable gastric banding operations [5]. In China, the number of bariatric operations has increased annually [6]. The standard set by the Chinese Society for Metabolic and Bariatric Surgery (CSMBS), which is adhered to by most Chinese bariatric surgeons, to determine patients who are qualified for weight loss surgery is as follows: (1) BMI ≥32.5 kg/m^2^ with or without type 2 diabetes mellitus (T2DM); (2) 27.5 kg/m^2^ <BMI <32.5 kg/m^2^ with T2DM but failed conservative treatment and combined with at least two metabolic diseases or comorbidities; (3) duration of T2DM ≤15 years with fasting C-peptide ≥50% of the normal lower limit; (4) waist circumference: male ≥90 cm, female ≥85 cm; and (5) age within 16∼65 years [6].

Although BMI is a common parameter used as an indication of overall adiposity, body fat distribution is considered to be a better indication of differential metabolic risks. Visceral adipose tissue (VAT), including perirenal, gonadal, epicardial, retroperitoneal, omental, and mesenteric adipose tissue, displays more adverse metabolic risk profiles, such as decreased insulin sensitivity and increased lipolytic activity, when compared with “healthy” subcutaneous adipose tissue (SAT) in an excessive energy state [7–9]. In addition, some proinflammatory cytokines have a predominant secretory pattern in VAT [9] under obese conditions. Recent studies have shown a link between race and gender with regard to adipose tissue storage [10]. Chinese people are more likely to develop central obesity when compared with Westerners [4], and the VAT of women is more strongly associated with metabolic factors than that of men [7]. Thus, there is an urgency to investigate the role of VAT in the incidence and development of obesity in Chinese women.

Proteins are executors of physiological functions and the direct embodiment of life phenomena. Focusing on the changes in VAT protein expression may bridge the gap between the regulation of gene expression and the signaling/metabolism changes in obesity. The proteomic approach, a reliable experimental approach, allows for global gene analysis at the protein level [11, 12]. However, most previous proteomic studies with regard to obesity have largely relied on experimental models and cultured adipocytes, and most of these studies used electrophoretic protein separation, which not only leads to limited proteome coverage but also results in a poor representation of low-abundance proteins or proteins with hydrophobicity [13]. In recent years, high-throughput proteomic techniques have enabled the reliable detection and quantitation of thousands of proteins in complex proteomes by liquid chromatography coupled to mass spectrometry (LC-MS). Despite their enormous potential, very few proteomic studies on human obesity and its comorbidities have applied these innovative approaches. In addition, limited studies have been conducted in Western populations. One LC-MS-based proteomic analysis discovered 39 plasma proteins that are longitudinally differential after weight loss and maintenance in nondiabetic overweight and obese European individuals [14]. To our knowledge, only one study has focused on the VAT proteome in human obesity. These authors found 250 proteins with significant abundance difference in age, T2DM, and gender comparisons in the Spanish population [13]. The lack of high-throughput studies on the adipose tissue protein composition in Eastern populations limits our understanding of the protein networks responsible for obesity and related metabolic responses in Easterners.

In this study, a high-throughput quantification method of label-free one-dimensional liquid chromatography tandem mass spectrometry (1D-LC-MS/MS) was used to compare the protein abundance of VAT between morbidly obese and normal weight Chinese women. This straightforward quantitative proteomics method is highly sensitive to MS analysis [15]. This analysis has both a higher dynamic range and proteome coverage capabilities compared with those of labeling techniques [16]. In theory, this method is scalable to any number of samples, especially complex samples [17]. Gene Ontology (GO) analysis and Ingenuity Pathway Analysis (IPA) were utilized in combination to analyze the biological functions and pathways in which these differential proteins are involved. Our current study on VAT proteomic profiles is an essential supplement to gain a clearer picture of VAT molecular processes in response to morbid obesity in Chinese women.

## 2. Materials and Methods

### 2.1. General Characteristics of the Study Subjects

A total of 9 morbidly obese female patients (mean age 33.80 ± 10.03 y; mean BMI 48.53 ± 9.68 kg/m^2^), who met the CSMBS standard for bariatric surgery, were recruited in this study and received laparoscopic adjustable gastric banding (LAGB). Eight normal weight females (mean age 44.13 ± 6.90 y; mean BMI 22.14 ± 1.55 kg/m^2^) who underwent elective abdominal surgical procedures (cholecystectomy, surgery for benign ovarian tumors, etc.) were included in this study as normal weight controls. All enrolled subjects were required to have normal hepatic-renal functions (ALT < 100 U/L, AST < 92 U/L, Cr < 132 *μ*mol/L, BUN < 7.14 mmol/L). Potential subjects with a history of cancer, acute inflammatory diseases, or any chronic diseases other than obesity and its comorbidities were excluded. Subjects who were taking weight loss pills, oral contraceptives, or other medications that could alter their lipid profiles or metabolic parameters were also excluded. A comprehensive nutritional assessment was performed before surgery. All participants reported that their body weight had been stable for at least three months. After a 10- to 12-hour overnight fast, blood samples were obtained from all participants and then measured by routine automated laboratory methods in our clinical laboratory. All enrolled patients also fasted overnight before the operation. This study was approved by the ethics committee of Peking Union Medical College Hospital, Beijing, China (Ethics Number: No. S-364). Informed written consent was obtained from all participants before the operation.

### 2.2. Preparation of the VAT Samples

VAT samples were extracted from similar anatomical locations in all subjects by experienced surgeons within 1 hour after opening the abdominal wall. These fresh VAT specimens were immediately washed with cold saline solution and then stored in liquid nitrogen at −80°C until analysis. The tissues were cut into small pieces, lysed in a buffer solution containing 7 M urea, 2 M thiourea, 65 mM dithioerythritol (DTE), and 83 mM Tris (Sigma-Aldrich, St. Louis, MO, USA) and homogenized with a homogenizer (IKA R104, IKA-Werke, Staufen, Germany) on ice. The extracts were then centrifuged at 20,000 *g* for 10 min at 4°C, and the supernatant was stored at −80°C. The protein contents of the adipose tissue samples were determined by a Bradford reagent kit (Thermo Fischer Scientific, MA, USA).

The total VAT proteins of 9 morbidly obese subjects were pooled, and the VAT proteins from 8 normal weight controls were also pooled at the same amount. The samples were then processed through columns (micro Biospin, Nanosep 10K Omega; PALL Co., Ann Arbor, MI, USA) according to the manufacturer's instructions [18]. This spin-filter aided method was used to ease low-abundance human protein identification. The samples were then deoxidized with 20 mM dithiothreitol (DTT), alkylated with 50 mM iodoacetamide (IAA), and digested with trypsin. After digestion, the peptides were desalted with Oasis HLB 3cc extraction cartridges (Oasis, Waters, Ireland), cleaned up with 500 *μ*L 0.1% formic acid, and eluted with 500 *μ*L 100% acetonitrile (ACN). Peptide elution was vacuum-dried and stored at −80°C.

### 2.3. 1D-Liquid Chromatography-Mass Spectrometry (1D-LC-MS/MS)

All eluted peptide samples from morbidly obese patients and normal weight controls were pooled into two groups. Each sample was analyzed by a RP C18 self-packing capillary LC column (75 *μ*m × 100 mm, 3 *μ*m). The eluted gradient was 5–30% buffer B1 (0.1% formic acid, 99.9% ACN; flow rate, 0.5 *μ*L/min) for 100 min. A Triple TOF 5600 was used to analyze the sample. The mass spectrometry data were acquired in the high-sensitivity mode using the following parameters: 30 data-dependent MS/MS scans per every full scan; full scans were acquired at resolution 40,000 and MS/MS scans at 20,000; 35% normalized collision energy, charge state screening (including precursors with +2 to +5 charge state), and dynamic exclusion (exclusion duration 15 s); the MS/MS scan range was 100–1800 *m/z*; and the scan time was 100 ms. We conducted three technical replicates (repetition test of mass spectrometry) to ensure system stability [19].

### 2.4. Proteomic Analysis

All MS/MS data were searched against the SwissProt human database from the UniProt website (http://www.uniprot.org) using Mascot (Matrix Science, London, UK; Version 2.3.02). The parameters were set as follows: the digestion enzyme end was semitrypsin; the precursor and fragment ion mass tolerances were 10 ppm and 0.8 Da, respectively; the carbamidomethyl of cysteine was a fixed modification; and 2 mis-cleavage sites were allowed.

The acquired wiff files were imported into Progenesis software (Nonlinear Dynamics, Version 4.0) for label-free quantification analysis. A reference run was automatically selected from the imported runs by Progenesis, and other runs were aligned to the reference run. Alignment was achieved by the automated alignment algorithm within the program. The features were filtered for charge state. To integrate the MS features with protein identification, the MS/MS spectra from all of the runs were exported and searched against the SwissProt human database by Mascot. The results were imported back into Progenesis, and the identified peptides were mapped to the quantitative peptide data. The peptide quantitative results were converted into protein quantitative results by peptide reassignment in Progenesis. Proteins with a fold change ≥2 and a *P* value <0.05 were considered statistically significant. Fold change refers to the ratio of the intensity of VAT protein expression in morbidly obese to normal weight women.

All differential proteins were assigned gene symbols via the Panther database (Protein Analysis through Evolutionary Relationships, http://www.pantherdb.org/). These differentially expressed proteins were further classified by the GO analysis (http://geneontology.org/). This classification system was to obtain functional annotations in categories of cellular component, molecular function, and biological process. Pathway analysis was performed on all differentially expressed proteins. For this purpose, the SwissProt accession numbers were entered into IPA software (http://www.ingenuity.com, Ingenuity Systems, Mountain View, CA, USA), which uses the protein location within cellular compartments to classify gene products and suggest possible biochemical, molecular, and biological functions. The differentially expressed proteins were also mapped to disease and function categories and canonical pathways, ranked by *z*-score and *P* value, respectively.

### 2.5. Western Blot Validation

The VAT proteins of 6 morbidly obese and 7 normal weight patients were extracted using a Total Protein Extraction kit (Applygen, Beijing, China), and the protein contents were determined using a Coomassie Protein Kit (Applygen). The total protein concentration was approximately 2 *μ*g/*μ*L. The proteins were then separated by SDS-PAGE and transferred onto nitrocellulose membranes (Millipore, MA, USA) using a wet transfer system (BIO-RAD, CA, USA). The membranes were blocked with 5% nonfat milk diluted with Tris-buffered saline/Tween-20 (TBST) (Jiangchenyuanyuan Biotechnology, Beijing, China) and then incubated with primary antibodies including anti-ACSL1 (Santa Cruz Biotechnology, CA, USA), anti-HADH (Santa Cruz Biotechnology), anti-UCHL1 (Santa Cruz Biotechnology), and anti-*β*-actin (Sigma-Aldrich) at dilutions ranging from 1 : 1000 to 1 : 4000 in 5% nonfat milk at 4°C overnight. After washing three times with TBST, the blots were further incubated with horseradish peroxidase- (HRP-) conjugated goat antibodies (Applygen) at a dilution of 1 : 1000 at room temperature for 1.5 hours. The specific protein bands were visualized by enhanced chemiluminescence (ECL) according to the manufacturer's instructions (Applygen). The bands were then quantified by Quantity One software (Version 4.6.9, Bio-Rad). *β*-Actin was used as an internal control for target protein analyses. Three independent experiments were carried out for all proteins.

### 2.6. Morphometry and Histological Analysis

Visceral adipose biopsies from 9 morbidly obese and 8 normal weight patients were dehydrated, paraffin embedded, and then sectioned (approximately 5 microns thick). The sections were stained with H&E and Masson's trichrome for histomorphology observation and collagen fiber evaluation. Immunohistochemistry was also performed after deparaffinization. Primary mouse anti-CD68 antibody (KP1) (ab955, Abcam, Cambridge, UK) was added and incubated overnight at 4°C for the identification of CD68 + macrophages. After washing 3 times with PBS, the sections were incubated with a goat anti-mouse horseradish peroxidase- (HRP-) conjugated secondary antibody (Vector Laboratories, CA, USA) for 1 hour. A diaminobenzidine color development kit (ZLI-9018, Zhong Shan Jin Qiao Company, Beijing, China) was used for the subsequent color development. The sections were visualized under a DM3000 Leica biological microscope.

### 2.7. Workflow of the Study

**Figure 1:**
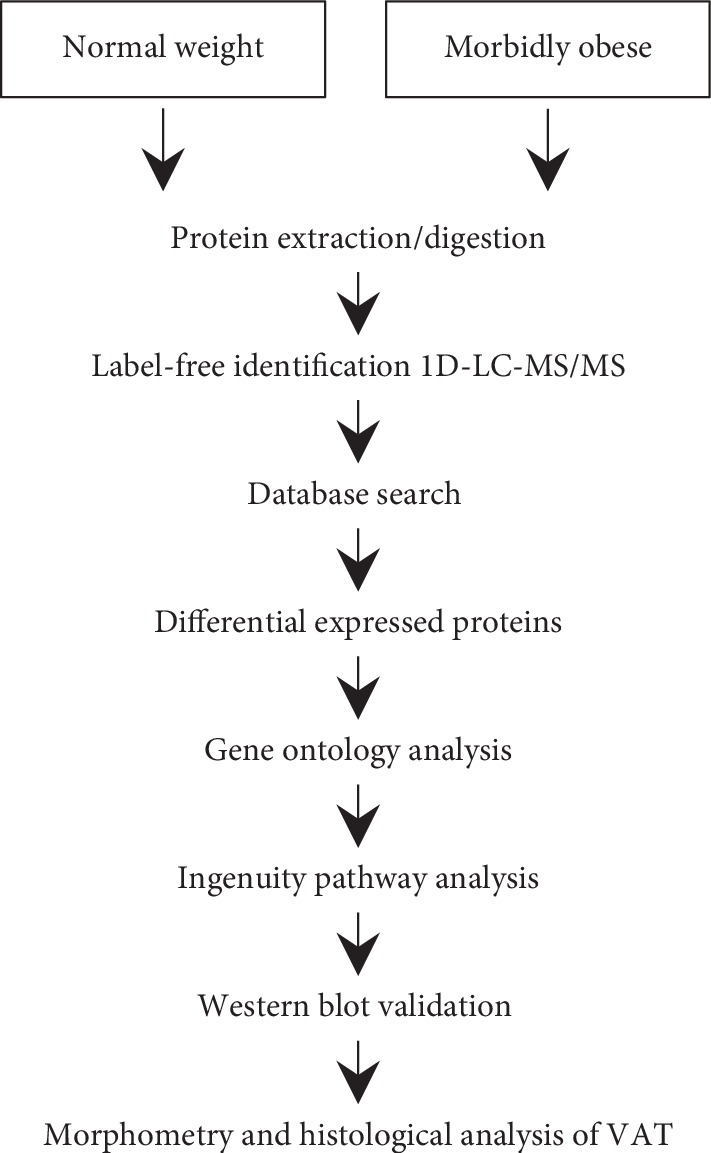
Workflow of the label-free 1D-LC/MS/MS proteomic strategy. In this work, visceral adipose tissue samples of 9 morbidly obese Chinese women and samples of 8 normal weight controls were extracted, pooled, and digested. The peptides were then sequenced using 1D-LC/MS/MS and label-free proteomic analysis. Technical replicates of mass spectrometry measures were conducted three times. GO analysis and IPA were used to analyze biological functions and pathways. Three candidate proteins were selected and further validated by western blotting analysis. Morphometry and immunohistochemical analyses were conducted between the two groups. Abbreviations: GO, Gene Ontology. IPA, Ingenuity Pathway Analysis.

Samples of VAT from both morbidly obese and normal weight groups were extracted, pooled, digested, and quantified via label-free 1D-LC-MS/MS. Technical replicates were obtained by three repeated mass spectrometry measures. Differentially expressed proteins from VAT proteomes were identified and further analyzed in conjunction with GO database and IPA. Three differentially expressed proteins were further validated by western blotting. Morphometry and histological analyses of visceral adipose tissue were conducted in both morbidly obese and normal weight groups. The workflow of this study is shown in Figure 1.

### 2.8. Statistical Methods

We performed statistical analysis with statistical software (SPSS 22.0, SPSS Inc. Chicago, IL) and performed Student's *t*-test or the Mann–Whitney *U*-test for group comparisons. Pearson's correlation coefficients were conducted to represent the relationships between western blotting results and anthropometric/laboratory measurements. Normally distributed data are presented as the mean ± SD. Nonnormally distribution parameters are reported as medians (1st quartile to 3rd quartile). *P* values less than 0.05 were considered to indicate a statistically significant difference.

## 3. Results

### 3.1. Anthropometric and Laboratory Measurements of Morbidly Obese and Normal Weight Subjects

Anthropometric and laboratory measurements of morbidly obese female subjects and normal weight controls are shown in Table 1. As expected, obese women had higher SBP and DBP, in addition to significantly higher body weight, BMI, and waist circumference than those of the normal weight controls (all *P* < 0.05). The mean age of obese women was lower than that of normal weight controls (*P* < 0.05). Metabolic profile-related blood work included fasting blood glucose, total cholesterol (TC), triglyceride (TG), high-density lipoprotein cholesterol (HDL-C), and low-density lipoprotein cholesterol (LDL-C). There was no significant difference in fasting blood glucose, TC, and LDL-C between the two groups. The TG level was higher while that of HDL-C was lower in morbidly obese women. As an inflammatory marker, the circulating hypersensitive C-reactive protein (hs-CRP) levels of morbidly obese subjects were much higher than the upper normal limit (3 mg/L). For normal weight controls, the hs-CRP levels were not available.

### 3.2. Protein Expression Profiles of VAT in Morbidly Obese and Normal Weight Subjects

**Figure 2:**
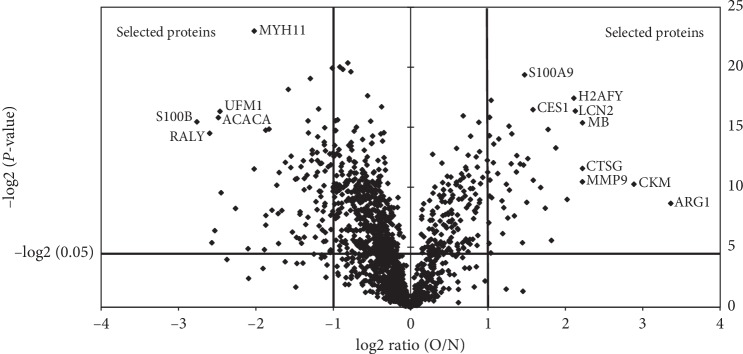
Volcano plot of the protein differences. The volcano plot illustrates the protein differences. The significantly expressed proteins are located above the horizontal line and on the outer side of the vertical lines. In this volcano plot, the horizontal line represents the 0.05 nominal significance level for protein expression. Vertical lines sort out proteins with at least a twofold difference between morbidly obese and normal weight subjects. Some most significantly changed proteins were identified and named in the figure. Abbreviations: O/N: obese/normal weight; MYH11, myosin-11; UFM1, ubiquitin-fold modifier 1; ACACA, acetyl-CoA carboxylase 1; S100B, protein S100-B; RALY, RNA-binding protein Raly; S100A9, protein S100-A9; H2AFY, core histone macro-H2A; CES1, liver carboxylesterase 1; LCN2, neutrophil gelatinase-associated lipocalin; MB, myoglobin; CTSG, cathepsin G; MMP9, matrix metalloproteinase-9; CKM, creatine kinase M-type; ARG1, arginase-1.

Using 1D-LC-MS/MS, we identified 1505 proteins across replicate experiments with a false positive rate of less than 1% (Supplement [Supplementary-material supplementary-material-1]). The coefficient of variation (CV) was then calculated to evaluate the technical variability across three run assays. Proteins with CV values at the top 5% were excluded. A total of 1374 proteins were finally obtained for further quantitative analysis. As shown in Figure 2, proteins with *P* values <0.05 and fold changes ≥2 (fold change is the ratio of intensity of protein expression) were selected. Some most significantly changed proteins were identified and named in this figure. These annotated proteins include myosin-11 (MYH11), ubiquitin-fold modifier 1 (UFM1), acetyl-CoA carboxylase 1 (ACACA), protein S100-B (S100B), RNA-binding protein Raly (RALY), protein S100-A9 (S100A9), core histone macro-H2A (H2AFY), liver carboxylesterase 1 (CES1), neutrophil gelatinase-associated lipocalin (LCN2), myoglobin (MB), cathepsin G (CTSG), matrix metalloproteinase-9 (MMP9), creatine kinase M-type (CKM), and arginase-1 (ARG1). The proteomic data and protein analysis information are listed in [Supplementary-material supplementary-material-1]. A total of 124 VAT proteins were considered to be differentially expressed, whereby 83 proteins were downregulated and 41 proteins were upregulated in obese individuals. Proteins with a significantly changed ratio (the top 20 downregulated differential proteins and the top 10 upregulated differential proteins) are shown in Table 2. All the 124 differential proteins are listed in Supplement Table S2.

### 3.3. Gene Ontology Analysis of the Differentially Expressed Proteins

**Figure 3:**
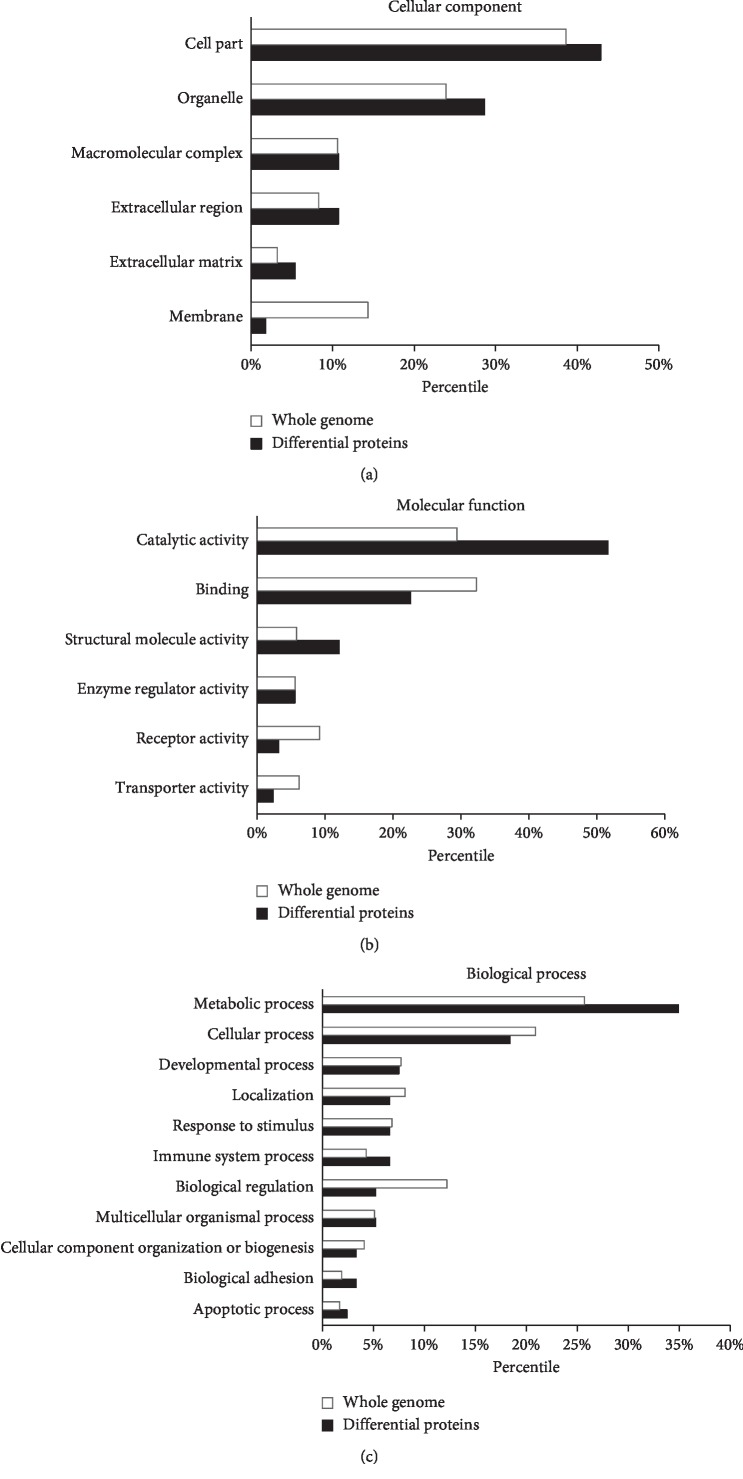
Gene Ontology analysis of the differentially expressed proteins. (a) Cellular component of the whole genome and the differentially expressed proteins in VAT. (b) Molecular function assigned to the whole genome and the differentially expressed proteins in VAT. (c) Biological processes assigned to the whole genome and the differentially expressed proteins in VAT.

The differentially expressed proteins identified in this study were classified in categories of cellular component, molecular function, and biological process by the GO database as presented in Figure 3. In the cellular component category, the differential proteins were mainly located in the organelles and cell parts (Figure 3(a)). When compared with the whole genome, the differentially expressed proteins involved in the catalytic activity and structural molecule activity were in high numbers, while proteins related to receptor activity and transporter activity were in relatively low numbers (Figure 3(b)). The differential proteins related to metabolic process, immune system process, etc., were overrepresented, whereas proteins related to cellular process, localization, etc., were underrepresented (Figure 3(c)).

### 3.4. Ingenuity Pathway Analysis of the Differentially Expressed Proteins

**Figure 4:**
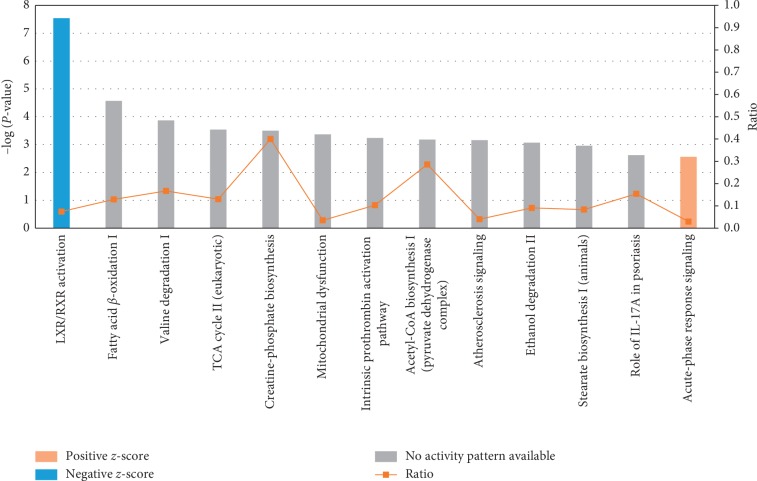
Ingenuity Pathway Analysis of the differentially expressed proteins. Differentially expressed proteins in VAT were characterized, and the top 13 canonical pathways were exhibited. Acute-phase response signaling was activated (red histogram). The LXR/RXR signaling pathway was inhibited (blue histogram).

IPA was applied to group the differentially expressed proteins on the basis of canonical pathways. The top 13 canonical pathways induced by the altered proteins include LXR/RXR activation, fatty acid *β*-oxidation I, valine degradation I, TCA cycle II (eukaryotic), creatine-phosphate biosynthesis, mitochondrial dysfunction, intrinsic prothrombin activation pathway, acetyl-CoA biosynthesis I (pyruvate dehydrogenase complex), atherosclerosis signaling, ethanol degradation II, stearate biosynthesis I (animals), role of IL-17A in psoriasis, and acute-phase response signaling (Figure 4). Among these pathways, LXR/RXR activation was attenuated, while acute-phase response signaling was enhanced, as indicated by the *z*-score. Other pathways did not show activation or attenuation. The network of the LXR/RXR activation pathway and the acute-phase response signaling pathway is shown in Supplement [Supplementary-material supplementary-material-1] and Supplement [Supplementary-material supplementary-material-1], respectively. As shown in Table 3, significantly changed proteins participating in these pathways include Complement 4A/Complement 4B (C4A/C4B), Lysozyme C (LYZ), Mitochondrial Enoyl-CoA Hydratase (ECHS1), and Apolipoprotein B-100 (APOB), etc.

### 3.5. Validation of Proteomic Results by Western Blotting

**Figure 5:**
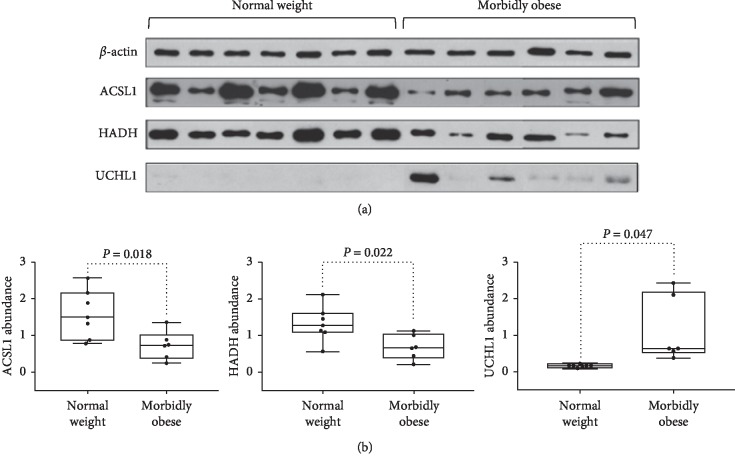
Western blot analysis of ACSL1, HADH, and UCHL1. (a) Three differential VAT proteins (HADH, ACSL1, and UCHL1) were validated by western blotting using the same set of VAT samples from 6 morbidly obese and 7 normal weight patients. (b) The box plots showed the density values from western blotting results between morbidly obese and normal weight groups.

To confirm proteomic changes in morbid obesity, the expression of three differential proteins (HADH, ACSL1, and UCHL1) was validated by western blot analysis using the same set of VAT samples from 6 obese and 7 normal weight patients with beta-actin as the internal control. As shown in Table 4 and Figure 5, the protein levels of ACSL1 and HADH in VAT of the obese patients were significantly decreased to 40% and 45%, respectively, compared with that of normal weight controls (*P* < 0.05). This result was in line with the observations in proteomic studies. Western blotting analysis was also performed to explore the expression of UCHL1, which showed that the expression of UCHL1 in obese VAT samples was upregulated in abundance when compared to that in normal weight controls with a fold change of 5.99. The western blotting results are consistent with the results obtained by label-free proteomic analysis methods.

### 3.6. Morphometry and Histological Analysis of VAT in Normal Weight and Morbidly Obese Subjects

**Figure 6:**
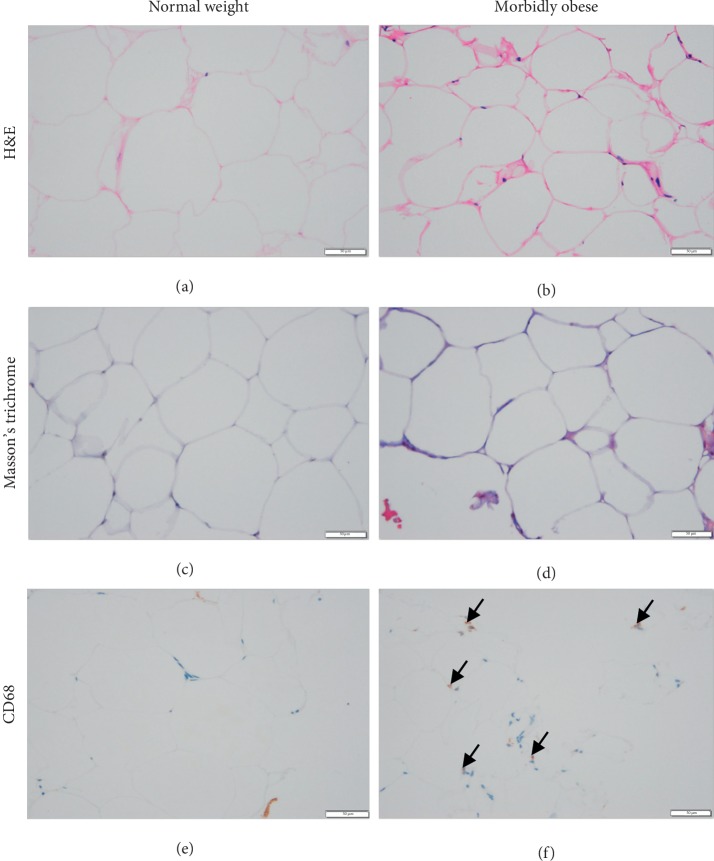
Morphometry and histological analysis of VAT in normal weight and morbidly obese subjects. (a, b) H&E staining showed that there was more inflammatory cellular infiltration in the VAT of morbidly obese patients. (c, d) Masson's trichrome staining demonstrated increased collagen fiber deposition (blue) in VAT of morbidly obese patients. (e, f) Representative immunohistochemical staining showed that there were more CD68-positive macrophages in the VAT of morbidly obese subjects. Arrowhead: macrophage. Scale bar: 50 *μ*m.

As displayed in Figure 6, representative H&E staining showed more inflammatory cellular infiltration in the VAT of the morbidly obese patients compared with that of controls. Masson's trichrome staining demonstrated increased collagen fiber deposition (blue) in the VAT of morbidly obese patients. Immunohistochemical staining showed that there were more CD68-positive macrophages in the VAT of morbidly obese patients than in controls.

## 4. Discussion

Fueled by the great hazard obesity poses, there is considerable interest in obesity-related studies. Considering the large proportion of abdominal obese individuals in China [4] and the lack of proteomic studies to analyze the adipose tissue of the Eastern population, we thus focused on differentially expressed proteins in VAT between morbidly obese and normal weight Chinese women to extend the understanding of the protein networks responsible for obesity in Easterners. By using label-free 1D-LC-MS/MS, we revealed 41 proteins with at least a 2-fold upregulation and 83 proteins with at least a 2-fold downregulation in morbidly obese subjects. With the help of GO analysis and IPA, we found that these differentially expressed VAT proteins contribute to the intensification of acute-phase response signaling and the attenuation of the LXR/RXR pathway. Three proteins, ACSL1, HADH, and UCHL1, were validated by western blotting methods, and the results show that the magnitude and direction of protein changes were in accordance with proteomic analysis. Morphometry and immunohistochemical analysis showed that the VATs of morbidly obese patients manifested increased inflammatory cell infiltration (including CD68-positive macrophages) and enhanced collagen fiber deposition. These morphological changes support our proteomic results.

### 4.1. LXR/RXR Signaling and Lipid Metabolic Disturbance

According to the IPA results, LXR/RXR activation was attenuated in VAT of morbidly obese Chinese female patients. LXR (the liver X receptor) exists in two isoforms, *α* and *β*, and these proteins combine with retinoid X receptor (RXR) and form the LXR/RXR heterodimer, which induces the transcription of multiple genes involved in cholesterol efflux, conversion, and transport [20] (related pathway is shown in [Supplementary-material supplementary-material-1]). This effect allows the regulation of the intracellular lipid surplus caused by a sedentary lifestyle and/or excessive caloric consumption [8]. Studies performed by Doumatey et al. also described the enrichment of the LXR/RXR pathway in the serum of metabolically unhealthy obese African American women by using proteomic analysis [21]. Apart from the circulatory system, both LXR*α* and LXR*β* are expressed in mature murine and human adipocytes [22]. MRI analysis revealed that the pharmacological activation of LXR modifies fat distribution by decreasing visceral fat [23]. Laffitte et al. demonstrated that the nuclear receptor LXR is a beneficial cholesterol sensor in adipose tissue that induces the transcription of genes that protect cells from cholesterol overload [24]. LXR*α*-knockout mice tend to gain more weight and accumulate fat mass on a high-fat diet compared with the effects on wild-type controls [25]. Despite these current studies, the role of LXR in adipose tissue is not very well confirmed. Our study suggests that there may exist a downshift in LXR/RXR activation in the VAT of morbidly obese Chinese women. Moreover, as shown in Table 3, LXR/RXR pathway attenuation is somehow related to the upregulation of C4A/C4B, LYZ, APOB, S100A8, and MMP9 and the downregulation of ECHS1, FASN, ACACA, and HADH in VAT. These results suggest that the liver X receptor pathway and related proteins could be potential treatment targets for obesity.

UCHL1 could promote the breakdown of RXR [26]. In this study, we found that the expression of UCHL1 is upregulated in the VAT of obese women by both proteomics and western blot analysis, which further helps explain the attenuation of the LXR/RXR pathway in VAT.

### 4.2. Inflammation and Acute-Phase Signaling Enhancement

Obesity is a metabolic problem and a chronic inflammatory disease. The level of circulating hs-CRP, an inflammatory biomarker, was elevated above the upper normal limit in our obese patient cohort (as shown in Table 1). However, the origin of inflammation during obesity and the underlying molecular mechanisms are still not fully understood. One explanation is that the systemic inflammation status is conditioned by acute-phase response, characterized by the elevation of acute-phase response protein levels [27]. With the blockade of one type of acute-phase protein, mice that received a high-fat diet were prevented from gaining weight and developed insulin resistance compared with controls [28]. At the protein level, serum proteomic analysis proved that metabolically unhealthy African American obese females suffered from abnormal enhancement of the acute-phase response signaling pathway [21]. A study of preobese diabetic subjects also showed that the proteins involved in inflammatory/immune responses were enriched in the VAT [29]. Consistent with previous studies, we also found that the acute-phase response signaling pathway is enhanced in the VAT of obese female patients (related pathway is shown in [Supplementary-material supplementary-material-1]).

The LXR/RXR signaling pathway exerts its anti-inflammatory effects by attenuating the release of inflammatory cytokines in the fat cells and macrophages of adipose tissue [30]. According to our study, LXR/RXR activation was attenuated in the VAT of obese female patients, suggesting that the downregulation of the LXR/RXR pathway may contribute to the enhancement of systemic inflammation in the VAT of obese patients.

### 4.3. Validation of Differential Proteins

Although differential proteomics research methods can reveal a large number of differentially expressed proteins, due to the limitation of the validation method, only a small amount of proteins could be verified. In this study, IPA eased the identification of obesity markers, and we chose ASCL1, HADH, and UCHL1 for validation based on both our proteomic analysis and previous research.

ACSL1, long-chain-fatty-acid-CoA ligase 1, belongs to the class of acyl-CoA synthetases, which are essential for the activation, transport, and degradation of fatty acids, as well as lipid synthesis [31]. According to our proteomic results, ACSL1 plays a role in fatty acid *β*-oxidation, one of the top canonical pathways. ACSL1 is localized to glucose transporter 4- (GLUT4-) containing vesicles [32]. ACSL1 deficiency impairs fasting glucose homeostasis in muscle [33] and downregulates the amount of cellular lipids and glucose uptake in adipocytes [34]. In adipose tissue, ACSL1 is one of the most highly expressed acyl CoA synthetases involved in the uptake of fatty acids and in triacylglycerol synthesis [35, 36]. Selective ACSL1 deficiency in mice caused an 80% loss of adipose ACSL activity [37]. Maternal obesity showed the decreased expression of ACSL1 in adipose tissue [38]. Moreover, ACSL1 polymorphisms confer a higher risk for pre-diabetes mellitus (pre-DM). Such pre-DM risk diminishes with decreasing BMI and disappears in subjects with normal weight (BMI less than 25 kg/m^2^) [39]. Since ACSL1 participates in various aspects of energy metabolism, we chose ACSL1 for validation. In the present study, both proteomics and western blotting results showed that ACSL1 expression in VAT was downregulated in morbidly obese Chinese women compared with normal weight controls. The downregulation of ACSL1 in VAT may indicate metabolic deterioration in the obese state.

HADH, hydroxyacyl-coenzyme A dehydrogenase, is a mitochondrial-oxidation enzyme of fatty acids and is widely involved in energy production, especially in periods of fasting and other metabolic stresses. HADH participates in the regulation of glucose-induced insulin release by normal beta cells, and it has also been associated with the K + ATP-independent regulation of insulin release by insulin-producing cells [40]. A loss-of-function mutation in the HADH gene was associated with fasting hyperinsulinemia [41, 42]. HADH is also related to the development of obesity and lipid metabolism. In an animal model, HADH (−/−) knockout mice showed that triglycerides were unable to be eliminated from the plasma during cold exposure compared with HADH (+/+) mice [43]. Another animal proteomic analysis revealed that differences in lipid deposition were regulated by HADH [44]. In our current study, HADH was revealed by IPA to participate in the LXR/RXR pathway (as shown in Table 3), and its downregulation may be associated with LXR/RXR pathway attenuation. Overall, the multiple roles of HADH in metabolism and its possible involvement in the LXR/RXR signaling pathway led us to validate HADH.

UCHL1, a ubiquitin carboxyl-terminal hydrolase isozyme L1, has been shown to be a fetal programming-related obesity marker [45] and could reflect the metabolic response of the inflammation state [46]. UCHL1 has been found to be involved in oxidative stress in adipose tissue [47]. Oxidative stress is considered to be one of the main factors that triggers and maintains inflammatory processes and leads to chronic, low-grade inflammation in obese patients [48]. Subcutaneous adipose tissue from obese males showed an upregulated pattern of UCHL1 [48]. UCHL1 could promote the breakdown of RXR [26]. Furthermore, increased UCHL1 expression and RXR*α* proteasomal degradation could be induced in vitro by conditions mimicking hypoxia, a condition that occurs in obese visceral adipose tissue [26]. These previous results are in line with our current one that VAT is manifested with UCHL1 upregulation and LXR/RXR signaling attenuation, which may indicate a harmful effect in enhanced oxidative stress in VAT in obese conditions. Fortunately, another study proposed that both short- and long-term weight loss could lead to downregulation of UCHL1 [49], which supports UCHL1 as a target for morbid obesity treatment. Due to the inflammatory involvement and therapeutic potential of UCHL1, we chose this protein for further validation.

We further conducted correlation analysis using western blotting results and clinical profiles (Supplement [Supplementary-material supplementary-material-1]). Both HADH and ACSL1 are positively correlated with HDL-C. ACSL1 is negatively correlated with TG, while UCHL1 is positively correlated with TG. These results indicated that differentially expressed VAT proteins are related to systemic dyslipidemia and metabolic disorders. In addition, the expression of ACSL1, HADH, and UCHL1 by western blotting was not correlated with age.

### 4.4. Adipocyte Morphometry, Fibrosis, and Inflammation Alterations

Our proteomic study revealed acute-phase response signaling enhancement and LXR/RXR signaling attenuation in VAT with regard to morbid obesity. Previous study has shown that adipose tissue macrophages are the major sources of obesity-associated inflammation [50]. In addition, LXR-dependent gene expression is important for macrophage survival and the innate immune response [51]. We conducted an immunohistochemistry study to investigate macrophage expression in VAT in obese patients, and we found that CD68, a marker of the macrophage lineage, was present at a higher expression level around adipocytes. This finding is a straightforward sign of increased macrophage involvement in the state of obesity and conforms to the signaling alterations in our study.

Chronic inflammation may activate tissue fibrosis, which becomes pathogenic, resulting in changes in the normal tissue structure and function [52]. Recently, increased human adipose tissue fibrosis has been associated with obesity and insulin resistance [53]. We also observed that some proteins, such as S100A8 and MMP9, are differentially expressed according to our proteome results. According to previous studies, S100A8 could promote fibroblast activation and fibrosis [54]. MMPs, a protein family that cleaves collagenous proteins, can regulate the dynamics of fibrosis by enabling remodeling of the extracellular matrix [55]. Thus, the inflammatory signaling changes as well as differentially altered proteins were hypothesized to affect collagen fiber deposition in VAT in our obese patients. After Masson's trichrome staining, abnormal collagen deposition, a hallmark of fibrosis development, was indeed observed in the VAT of morbidly obese patients.

Altogether, inflammation and tissue fibrosis seem to go hand in hand and are associated with VAT proteomic changes under the condition of morbid obesity.

### 4.5. Limitation

The primary limitation of this study is the reduced number of patients finally enrolled, which is partly due to the recruit difficulty considering the homogeneous clinical features. Owing to the difficulties inherent to obtaining patient tissue samples, most previous clinical proteomics studies were also based on relatively small patient cohorts [56, 57]. We tried to lessen the detrimental effect of the relatively limited number of samples by the application of a robust proteomic procedure and technical replicates [58].

The mismatch of age is another limitation of this study. The age of the control subjects was relatively higher. In animal models, age-related proteomic changes are minor in lean mice [59]. Further statistical correlation analysis between western blotting results and age was conducted, and the results showed that the expression of ACSL1, HADH, and UCHL1 was not correlated with age ([Supplementary-material supplementary-material-1]).

## 5. Conclusion

In this study, we observed 124 differentially expressed proteins in the VAT of morbidly obese Chinese females compared with normal weight subjects. Eighty-three of these proteins were downregulated, while 41 proteins were upregulated. By further analysis, we found that these altered VAT proteins may be involved in the attenuation of the liver X receptor/retinoid X receptor (LXR/RXR) signaling pathway as well as the activation of the acute-phase response process. The western blotting results were in accordance with proteomic analysis for protein changes. Morphometry and histological analyses revealed that the VATs of morbidly obese subjects manifested increased inflammatory cell infiltration (including CD68-positive macrophages) and enhanced collagen fiber deposition. These identified differentially expressed proteins could potentially be targets in addressing the role of VAT in the state of morbid obesity.

## Figures and Tables

**Table 1:** Anthropometric and laboratory measurements of morbidly obese and normal weight subjects.

	Morbidly obese (*N* = 9)	Normal weight (*N* = 8)	*P* value
Age (years)	33.80 ± 10.03	44.13 ± 6.90	0.015
Height (m)	1.63 ± 0.05	1.64 ± 0.05	0.505
Weight (kg)	128.44 ± 26.95	59.81 ± 4.85	0.000
BMI (kg/m^2^)	48.53 ± 9.68	22.14 ± 1.55	0.000
WC (cm)	133.72 ± 16.59	73.88 ± 2.42	0.000
SBP (mmHg)	140.00 (129.50–150.00)	110.00 (104.50–113.50)	0.006
DBP (mmHg)	90.00 (82.00–102.50)	67.50 (60.75–79.25)	0.006
FBG(mmol/L)	7.28 ± 2.90	5.24 ± 0.30	0.069
TC (mmol/L)	5.12 ± 0.39	4.69 ± 0.53	0.583
TG (mmol/L)	1.93 ± 0.24	0.68 ± 0.08	0.001
HDL-C (mmol/L)	1.02 ± 0.09	1.82 ± 0.38	0.017
LDL-C (mmol/L)	3.44 ± 0.29	2.36 ± 0.29	0.076
hs-CRP (mg/L)	10.00 (2.52–10.00)	—	—

Data are means ± SD. Nonnormally distribution parameters were reported as medians (1st quartile to 3rd quartile). Abbreviations: BMI, body mass index; WC, waist circumference; SBP, systolic blood pressure; DBP, diastolic blood pressure; FBG, fasting blood glucose; TC, total cholesterol; TG, triglyceride; HDL-C, high-density lipoprotein cholesterol; LDL-C, low-density lipoprotein cholesterol; hs-CRP, hypersensitive C-reactive protein.

**Table 2:** List of differentially expressed proteins with top changing ratio.

Swiss-prot accession number	Gene symbol	Name	Ratio (O/N)	Style
P04271	S100B	Protein S100-B	0.147332273	Down
Q86WU2	LDHD	Probable D-lactate dehydrogenase, mitochondrial	0.168476843	Down
Q13085	ACACA	Acetyl-CoA carboxylase 1	0.178573909	Down
P61960	UFM1	Ubiquitin-fold modifier 1	0.181188644	Down
P49327	FASN	Fatty acid synthase	0.18307047	Down
P24298	GPT	Alanine aminotransferase 1	0.208245037	Down
P35749	MYH11	Myosin-11	0.246284001	Down
P61604	HSPE1	10 kDa heat shock protein, mitochondrial	0.272970627	Down
P11498	PC	Pyruvate carboxylase, mitochondrial	0.273082068	Down
P51911	CNN1	Calponin-1	0.273354388	Down
Q9UD71	PPP1R1B	Protein phosphatase 1 regulatory subunit 1B	0.281236999	Down
P30837	ALDH1B1	Aldehyde dehydrogenase X, mitochondrial	0.288701859	Down
Q14117	DPYS	Dihydropyrimidinase	0.301388582	Down
O15075	DCLK1	Serine/threonine-protein kinase DCLK1	0.305100359	Down
P23297	S100A1	Protein S100-A1	0.307875276	Down
P99999	CYCS	Cytochrome c	0.315465993	Down
Q9NVH6	TMLHE	Trimethyllysine dioxygenase, mitochondrial	0.325505242	Down
P21589	NT5E	5′-nucleotidase	0.334139431	Down
P12277	CKB	Creatine kinase B-type	0.341811143	Down
P07451	CA3	Carbonic anhydrase 3	0.342863108	Down
P01766	Ig heavy chain V-III region BRO	Ig heavy chain V-III region BRO	2.014026303	Up
P02747	C1QC	Complement C1q subcomponent subunit C	2.019800839	Up
O76070	SNCG	Gamma-synuclein	2.026450854	Up
P04114	APOB	Apolipoprotein B-100	2.028866497	Up
P02751	FN1	Fibronectin	2.030956781	Up
P11277	SPTB	Spectrin beta chain, erythrocytic	2.034775214	Up
P02788	LTF	Lactotransferrin	2.057043651	Up
P61626	LYZ	Lysozyme C	2.067956606	Up
P01876	IGHA1	Ig alpha-1 chain C region	2.098518872	Up
P01877	IGHA2	Ig alpha-2 chain C region	2.105459061	Up

Abbreviations: O/N: obese/normal weight.

**Table 3:** Top canonical pathways and related differential proteins.

Ingenuity canonical pathways	−log (*P* value)	*z*-score	Proteins
LXR/RXR activation	7.54	−1.342	C4A/C4B, LYZ, ECHS1, APOB, FASN, ACACA, S100A8, MMP9, HADH
Fatty acid *β*-oxidation I	4.57	NaN	ECHS1, ECI1, ACSL1, HADH
Valine degradation I	3.87	NaN	ECHS1, BCKDHA, ALDH6A1
TCA cycle II (eukaryotic)	3.54	NaN	CS, SUCLG1, MDH2
Creatine-phosphate biosynthesis	3.50	NaN	CKB, CKM
Mitochondrial dysfunction	3.37	NaN	PDHA1, NDUFA4, CYCS, DHODH, ATP5I, UQCRB
Intrinsic prothrombin activation pathway	3.24	NaN	COL1A2, FGB, FGG
Acetyl-CoA biosynthesis I (pyruvate dehydrogenase complex)	3.18	NaN	PDHA1, PDHB
Atherosclerosis signaling	3.16	NaN	COL1A2, LYZ, APOB, S100A8, MMP9
Ethanol degradation II	3.07	NaN	ALDH1B1, ADH1B, ACSL1
Stearate biosynthesis I (animals)	2.96	NaN	FASN, ACOT1, ACSL1
Role of IL-17A in psoriasis	2.62	NaN	S100A9, S100A8
Acute-phase response signaling	2.56	2	C4A/C4B, FTL, FN1, FGB, FGG

*z*-score was to infer the activation state of pathways. Positive *z*-score represented that the pathway was activated; negative *z*-score indicated that the pathway was suppressed.

**Table 4:** Protein expression by proteomic and western blotting analysis.

Protein	Proteomic analysis	Western blot analysis	*P* value
	Fold change (O/N)	Fold change (O/N)	
ACSL1	0.41	0.40	0.018
HADH	0.41	0.45	0.022
UCHL1	2.70	5.99	0.047

*P* value scales the difference of western blotting results between the O/N group. Abbreviations: O/N: obese/normal weight; ACSL1, long-chain-fatty-acid-CoA ligase 1; HADH, hydroxyacyl-coenzyme A dehydrogenase; UCHL1, ubiquitin carboxyl-terminal hydrolase isozyme L1.

## Data Availability

The raw proteomic data used to support the findings of this study are included in the supplementary materials. The clinical data are included within the article.

## Supplementary Materials

Supplementary Materials Table S1: scaffold reports for proteins in VAT identified by label-free 1D-LC-MS/MS. Table S2: the 124 differentially expressed VAT proteins between morbidly obese and normal weight subjects. Figure S1: LXR/RXR signaling pathway with participating proteins. Figure S2: acute phase response signaling pathway with participating proteins. Table S3: correlation analysis of western blotting results and anthropometric/laboratory measurements.
