# Supplementary material for: Comparative Proteomic Analysis of Visceral Adipose Tissue in Morbidly Obese and Normal Weight Chinese Women
Source: Int J Endocrinol. 2019 Dec 18;2019:2302753. doi: 10.1155/2019/2302753 (PMC6935805; doi:10.1155/2019/2302753)
Supplement: Supplementary Materials — Table S1: scaffold reports for proteins in VAT identified by label-free 1D-LC-MS/MS. Table S2: the 124 differentially expressed VAT proteins between morbidly obese and normal weight subjects. Figure S1: LXR/RXR signaling pathway with participating proteins. Figure S2: acute phase response signaling pathway with participating proteins. Table S3: correlation analysis of western blotting results and anthropometric/laboratory measurements. [file 2302753.f1.zip › Supplementary Materials/Table S2.pdf]

Table S2 The 124 differentially expressed VAT proteins between morbidly obese and normal weight subjects

Swiss-prot accession number	Gene symbol	Name	Ratio(O/N)	Style
04271	S100B	Protein S100-B	0.147332273	down
Q86WU2	LDHD	Probable D-lactate dehydrogenase, mitochondrial	0.168476843	down
Q13085	ACACA	Acetyl-CoA carboxylase 1	0.178573909	down
P61960	UFM1	Ubiquitin-fold modifier 1	0.181188644	down
P49327	FASN	Fatty acid synthase	0.18307047	down
P24298	GPT	Alanine aminotransferase 1	0.208245037	down
P35749	MYH11	Myosin-11	0.246284001	down
P61604	HSPE1	10 kDa heat shock protein, mitochondrial	0.272970627	down
P11498	PC	Pyruvate carboxylase, mitochondrial	0.273082068	down
P51911	CNN1	Calponin-1	0.273354388	down
Q9UD71	PPP1R1 B	Protein phosphatase 1 regulatory subunit 1B	0.281236999	down
P30837	ALDH1 B1	Aldehyde dehydrogenase X, mitochondrial	0.288701859	down
Q14117	DPYS	Dihydropyrimidinase	0.301388582	down
O15075	DCLK1	Serine/threonine-protein kinase DCLK1	0.305100359	down
P23297	S100A1	Protein S100-A1	0.307875276	down
P99999	CYCS	Cytochrome c	0.315465993	down
Q9NVH6	TMLHE	Trimethyllysine dioxygenase, mitochondrial	0.325505242	down
P21589	NT5E	5'-nucleotidase	0.334139431	down
P12277	CKB	Creatine kinase B-type	0.341811143	down
P07451	CA3	Carbonic anhydrase 3	0.342863108	down
A4D1P6	WDR91	WD repeat-containing protein 91	0.34664244	down
Q01995	TAGLN	Transgelin	0.356222802	down
P30084	ECHS1	Enoyl-CoA hydratase, mitochondrial	0.361462798	down
Q96AB3	ISOC2	Isochorismatase domain-containing protein 2, mitochondrial	0.370891294	down

P08559	PDHA1	Pyruvate dehydrogenase E1 component subunit alpha, somatic form, mitochondrial	0.371565161	down
P24844	MYL9	Myosin regulatory light polypeptide 9	0.374458944	down
Q01469	FABP5	Fatty acid-binding protein, epidermal	0.376171355	down
Q16822	PCK2	Phosphoenolpyruvate carboxykinase GTP, mitochondrial	0.380963014	down
O00483	NDUFA4	NADH dehydrogenase ubiquinone 1 alpha subcomplex subunit 4	0.382424637	down
Q02252	ALDH6A1	Methylmalonate-semialdehyde dehydrogenase acylating, mitochondrial	0.385143747	down
P32455	GBP1	Interferon-induced guanylate-binding protein 1	0.38599138	down
Q63ZY3	KANK2	KN motif and ankyrin repeat domain-containing protein 2	0.39544228	down
P14927	UQCRB	Cytochrome b-c1 complex subunit 7	0.398552128	down
Q05707	COL14A1	Collagen alpha-1(XIV) chain	0.398758022	down
P56385	ATP5I	ATP synthase subunit e, mitochondrial	0.400206252	down
P42126	ECI1	Enoyl-CoA delta isomerase 1, mitochondrial	0.404033959	down
Q9HCC0	MCCC2	Methylcrotonoyl-CoA carboxylase beta chain, mitochondrial	0.407064319	down
Q16836	HADH	Hydroxyacyl-coenzyme A dehydrogenase, mitochondrial	0.4118854	down
P21291	CSRP1	Cysteine and glycine-rich protein 1	0.414037406	down
P33121	ACSL1	Long-chain-fatty-acid--CoA ligase 1	0.414279112	down
Q76LX8	ADAMTS13	A disintegrin and metalloproteinase with thrombospondin motifs 13	0.420590654	down
Q9BX68	HINT2	Histidine triad nucleotide-binding protein 2, mitochondrial	0.425592624	down

O43143	DHX15	Putative pre-mRNA-splicing factor ATP-dependent RNA helicase DHX15	0.427703843	down
Q7Z434	MAVS	Mitochondrial antiviral-signaling protein	0.428256467	down
Q8NC51	SERBP1	Plasminogen activator inhibitor 1 RNA-binding protein	0.432337657	down
Q96C23	GALM	Aldose 1-epimerase	0.432565543	down
Q6YN16	HSDL2	Hydroxysteroid dehydrogenase-like protein 2	0.434080867	down
Q13188	STK3	Serine/threonine-protein kinase 3	0.434094602	down
P55010	EIF5	Eukaryotic translation initiation factor 5	0.435053867	down
Q86TX2	ACOT1	Acyl-coenzyme A thioesterase 1	0.438509374	down
Q96DC8	ECHDC3	Enoyl-CoA hydratase domain-containing protein 3, mitochondrial	0.440493665	down
P53597	SUCLG1	Succinyl-CoA ligase ADP/GDP-forming subunit alpha, mitochondrial	0.441446251	down
P00325	ADH1B	Alcohol dehydrogenase 1B	0.444440705	down
P17931	LGALS3	Galectin-3	0.446655756	down
P16070	CD44	CD44 antigen	0.447567406	down
O43294	TGFB1I1	Transforming growth factor beta-1-induced transcript 1 protein	0.448881632	down
P62081	RPS7	40S ribosomal protein S7	0.45039184	down
P08195	SLC3A2	4F2 cell-surface antigen heavy chain	0.453354191	down
P68036	UBE2L3	Ubiquitin-conjugating enzyme E2 L3	0.453382906	down
Q92945	KHSRP	Far upstream element-binding protein 2	0.456573891	down
Q6P2I3	FAHD2B	Fumarylacetoacetate hydrolase domain-containing protein 2B	0.460174064	down
Q14980	NUMA1	Nuclear mitotic apparatus protein 1	0.460993453	down
P09493	TPM1	Tropomyosin alpha-1 chain	0.461845707	down

Q02127	DHOD H	Dihydroorotate dehydrogenase (quinone), mitochondrial	0.462273586	down
O43175	PHGDH	D-3-phosphoglycerate dehydrogenase	0.463186399	down
P68402	PAFAH 1B2	Platelet-activating factor acetylhydrolase IB subunit beta	0.463406178	down
O75208	COQ9	Ubiquinone biosynthesis protein COQ9, mitochondrial	0.464233254	down
P11177	PDHB	Pyruvate dehydrogenase E1 component subunit beta, mitochondrial	0.464631415	down
O95394	PGM3	Phosphoacetylglucosamine mutase	0.465669995	down
P09429	HMGB1	High mobility group protein B1	0.466191665	down
P13804	ETFA	Electron transfer flavoprotein subunit alpha, mitochondrial	0.467319105	down
Q7Z4W1	DCXR	L-xylulose reductase	0.478036298	down
P14174	MIF	Macrophage migration inhibitory factor	0.484755952	down
P41567	EIF1	Eukaryotic translation initiation factor 1	0.48589058	down
P12694	BCKDH A	2-oxoisovalerate dehydrogenase subunit alpha, mitochondrial	0.486778751	down
Q96FV2	SCRN2	Secernin-2	0.488518863	down
P05166	PCCB	Propionyl-CoA carboxylase beta chain, mitochondrial	0.488799632	down
O75390	CS	Citrate synthase, mitochondrial	0.4891423	down
Q8IY63	AMOT L1	Angiomotin-like protein 1	0.489881728	down
O15247	CLIC2	Chloride intracellular channel protein 2	0.495709921	down
Q14118	DAG1	Dystroglycan	0.497870577	down
P00966	ASS1	Argininosuccinate synthase	0.498373993	down
P40926	MDH2	Malate dehydrogenase, mitochondrial	0.498930212	down
P01766	Ig heavy chain V-III region BRO	Ig heavy chain V-III region BRO	2.014026303	up

P02747	C1QC	Complement C1q subcomponent subunit C	2.019800839	up
O76070	SNCG	Gamma-synuclein	2.026450854	up
P04114	APOB	Apolipoprotein B-100	2.028866497	up
P02751	FN1	Fibronectin	2.030956781	up
P11277	SPTB	Spectrin beta chain, erythrocytic	2.034775214	up
P02788	LTF	Lactotransferrin	2.057043651	up
P61626	LYZ	Lysozyme C	2.067956606	up
P01876	IGHA1	Ig alpha-1 chain C region	2.098518872	up
P01877	IGHA2	Ig alpha-2 chain C region	2.105459061	up
Q13620	CUL4B	Cullin-4B	2.182249748	up
P02679	FGG	Fibrinogen gamma chain	2.202826065	up
P51636	CAV2	Caveolin-2	2.267193486	up
Q13434	MKRN4P	Putative E3 ubiquitin-protein ligase makorin-4	2.281286946	up
P43626	KIR2DL1	Killer cell immunoglobulin-like receptor 2DL1	2.303471773	up
P05164	MPO	Myeloperoxidase	2.357126718	up
P02792	FTL	Ferritin light chain	2.388003798	up
P02675	FGB	Fibrinogen beta chain	2.414039455	up
P0C0L4	C4A	Complement C4-A	2.422223056	up
P01911	HLA-DRB1	HLA class II histocompatibility antigen, DRB1-15 beta chain	2.46175602	up
P01814	Ig heavy chain V-II region OU	Ig heavy chain V-II region OU	2.475359729	up
P04259	KRT6B	Keratin, type II cytoskeletal 6B	2.533243095	up
P0C0L5	C4B	Complement C4-B	2.579543693	up
Q9UIF7	MUTYH	A/G-specific adenine DNA glycosylase	2.588527071	up
P05109	S100A8	Protein S100-A8	2.668429849	up
P09936	UCHL1	Ubiquitin carboxyl-terminal hydrolase isozyme L1	2.749541842	up
P06702	S100A9	Protein S100-A9	2.774986439	up
P15559	NQO1	NAD(P)H dehydrogenase quinone 1	2.829538719	up
P08123	COL1A2	Collagen alpha-2(I) chain	2.857271519	up
P23141	CES1	Liver carboxylesterase 1	2.992110681	up

P08779	KRT16	Keratin, type I cytoskeletal 16	2.999859174	up
Q92781	RDH5	11-cis retinol dehydrogenase	3.204484525	up
Q13586	STIM1	Stromal interaction molecule 1	3.339665768	up
P02511	CRYAB	Alpha-crystallin B chain	3.424798843	up
P20160	AZU1	Azurocidin	3.530126213	up
Q969L2	MAL2	Protein MAL2	3.670527105	up
O75367	H2AFY	Core histone macro-H2A.1	4.314654909	up
P80188	LCN2	Neutrophil gelatinase-associated lipocalin	4.371978082	up
P14780	MMP9	Matrix metalloproteinase-9	4.658424541	up
P02144	MB	Myoglobin	4.661746864	up
P06732	CKM	Creatine kinase M-type	7.392532524	up

Abbreviations：O/N: Obese/ Normal weight
