# Supplementary material for: Comparative Proteomic Analysis of Visceral Adipose Tissue in Morbidly Obese and Normal Weight Chinese Women
Source: Int J Endocrinol. 2019 Dec 18;2019:2302753. doi: 10.1155/2019/2302753 (PMC6935805; doi:10.1155/2019/2302753)
Supplement: Supplementary Materials — Table S1: scaffold reports for proteins in VAT identified by label-free 1D-LC-MS/MS. Table S2: the 124 differentially expressed VAT proteins between morbidly obese and normal weight subjects. Figure S1: LXR/RXR signaling pathway with participating proteins. Figure S2: acute phase response signaling pathway with participating proteins. Table S3: correlation analysis of western blotting results and anthropometric/laboratory measurements. [file 2302753.f1.zip › Supplementary Materials/Table S3.pdf]

Table S3 Correlation analysis of western blotting results and anthropometric/ laboratory measurements

	Western blotting results					
	HADH		ACSL1		UCHL1	
	r	P value	r	P value	r	P value
**Age**	0.626	0.053	0.536	0.110	-0.066	0.857
**Height**	0.389	0.266	0.196	0.588	-0.251	0.484
**Weight**	-0.515	0.127	-0.380	0.278	0.313	0.378
**BMI**	-0.579	0.080	-0.422	0.225	0.362	0.305
**WC**	-0.636*	0.048	-0.454	0.188	0.432	0.212
**SBP**	-0.010	0.979	-0.048	0.896	0.417	0.230
**DBP**	-0.123	0.735	-0.169	0.640	0.563	0.090
**FBG**	-0.363	0.303	-0.426	0.219	0.445	0.197
**TC**	0.102	0.780	-0.034	0.925	0.148	0.684
**TG**	-0.568	0.087	-0.689*	0.027	0.853*	0.002
**HDL**	0.765*	0.010	0.715*	0.020	-0.310	0.383
**LDL**	-0.260	0.467	-0.357	0.311	0.176	0.627

r. Pearson's coefficient. *p value<0.05.

Abbreviations: BMI, body mass index; WC, waist circumference; SBP, systolic blood pressure; DBP, diastolic blood pressure; FBG, fasting blood glucose; TC, total cholesterol; TG, triglyceride; HDL-C, high density lipoprotein cholesterol; LDL-C, low density lipoprotein cholesterol; ACSL1, long-chain-fatty-acid-CoA ligase 1; HADH, hydroxyacyl-coenzyme A dehydrogenase; UCHL1, ubiquitin carboxyl-terminal hydrolase isozyme L1.
