# Supplementary material for: Comparative Proteomic Analysis of Visceral Adipose Tissue in Morbidly Obese and Normal Weight Chinese Women
Source: Int J Endocrinol. 2019 Dec 18;2019:2302753. doi: 10.1155/2019/2302753 (PMC6935805; doi:10.1155/2019/2302753)
Supplement: Supplementary Materials — Table S1: scaffold reports for proteins in VAT identified by label-free 1D-LC-MS/MS. Table S2: the 124 differentially expressed VAT proteins between morbidly obese and normal weight subjects. Figure S1: LXR/RXR signaling pathway with participating proteins. Figure S2: acute phase response signaling pathway with participating proteins. Table S3: correlation analysis of western blotting results and anthropometric/laboratory measurements. [file 2302753.f1.zip › Supplementary Materials/Figure S1.pdf]

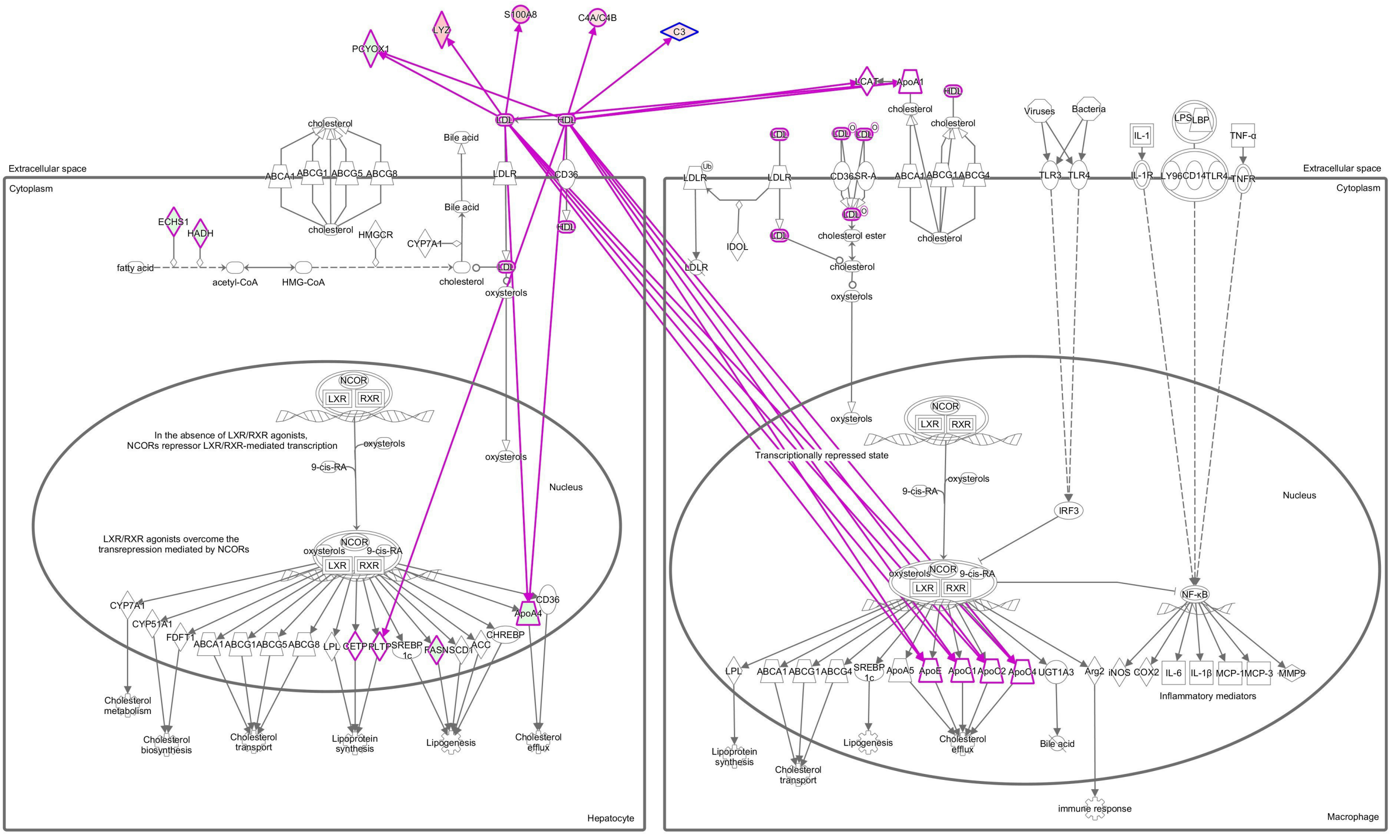

Figure S1 LXR/RXR signaling pathway with participated proteins

It shows the LXR/RXR signaling pathway network. Proteins in red color are upregulated in VAT in morbidly obese Chinese women. Proteins in green color are downregulated in VAT in morbidly obese Chinese women.
