# Supplementary material for: Comparative Proteomic Analysis of Visceral Adipose Tissue in Morbidly Obese and Normal Weight Chinese Women
Source: Int J Endocrinol. 2019 Dec 18;2019:2302753. doi: 10.1155/2019/2302753 (PMC6935805; doi:10.1155/2019/2302753)
Supplement: Supplementary Materials — Table S1: scaffold reports for proteins in VAT identified by label-free 1D-LC-MS/MS. Table S2: the 124 differentially expressed VAT proteins between morbidly obese and normal weight subjects. Figure S1: LXR/RXR signaling pathway with participating proteins. Figure S2: acute phase response signaling pathway with participating proteins. Table S3: correlation analysis of western blotting results and anthropometric/laboratory measurements. [file 2302753.f1.zip › Supplementary Materials/Figure S2.pdf]

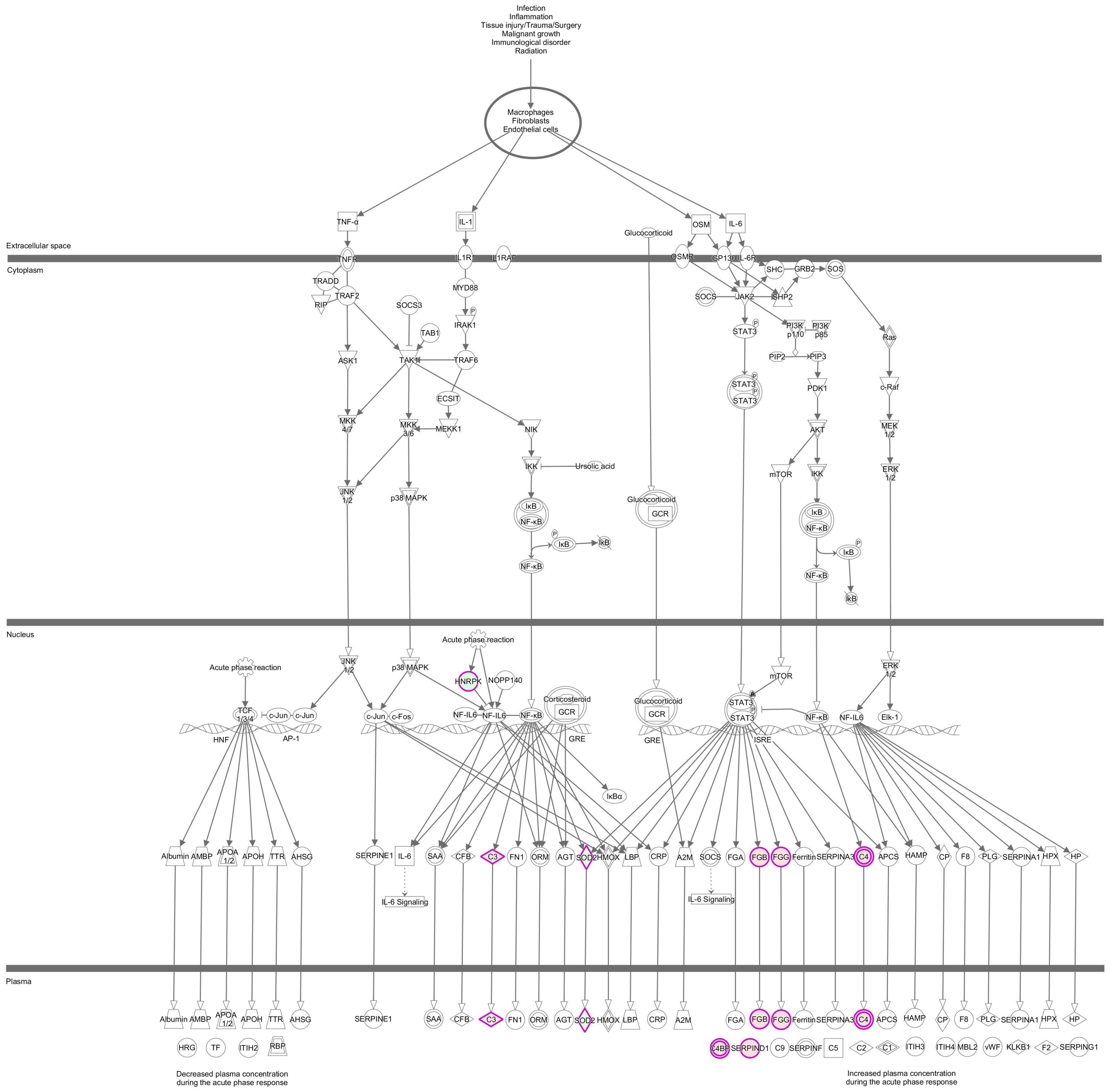

Figure S2 Acute phase response signaling pathway with participated proteins

It shows the acute phase response signaling pathway network. Proteins in red color are upregulated in VAT in morbidly obese Chinese women. Proteins in green color are downregulated in VAT in morbidly obese Chinese women.
